# Accuracy of in-utero MRI to detect fetal brain abnormalities and prognosticate developmental outcome: postnatal follow-up of the MERIDIAN cohort

**DOI:** 10.1016/S2352-4642(19)30349-9

**Published:** 2020-02

**Authors:** Anthony R Hart, Nicholas D Embleton, Michael Bradburn, Daniel J A Connolly, Laura Mandefield, Cara Mooney, Paul D Griffiths

**Affiliations:** a Department of Paediatric and Perinatal Neurology, Sheffield Children's Hospital NHS Foundation Trust, Ryegate Children's Centre, Sheffield, UK; b Newcastle Neonatal Service, Ward 35 Neonatal Unit, Royal Victoria Infirmary, Newcastle Hospitals NHS Foundation Trust, Newcastle upon Tyne, UK; c Clinical Trials Research Unit, School Health and Related Research, University of Sheffield, Sheffield, UK; d Department of Paediatric Neuroradiology, Sheffield Children's Hospital NHS Foundation Trust, Western Bank, Sheffield, UK; e Academic Unit of Radiology, University of Sheffield, Royal Hallamshire Hospital, Sheffield, UK

## Abstract

**Background:**

In utero MRI (iuMRI) detects fetal brain abnormalities more accurately than ultrasonography and provides additional clinical information in around half of pregnancies. We aimed to study whether postnatal neuroimaging after age 6 months changes the diagnostic accuracy of iuMRI and its ability to predict developmental outcome.

**Methods:**

Families enrolled in the MERIDIAN study whose child survived to age 3 years were invited to have a case note review and assessment of developmental outcome with the Bayley Scales of Infant and Toddler Development, the Ages and Stages Questionnaire, or both. A paediatric neuroradiologist, masked to the iuMRI results, reviewed the postnatal neuroimaging if the clinical report differed from iuMRI findings. Diagnostic accuracy was recalculated. A paediatric neurologist and neonatologist categorised participants' development as normal, at risk, or abnormal, and the ability of iuMRI and ultrasonography to predict developmental outcome were assessed.

**Findings:**

210 participants had case note review, of whom 81 (39%) had additional investigations after age 6 months. The diagnostic accuracy of iuMRI remained higher than ultrasonography (proportion of correct cases was 529 [92%] of 574 *vs* 387 [67%] of 574; absolute difference 25%, 95% CI 21 to 29; p<0·0001). Developmental outcome data were analysed in 156 participants, and 111 (71%) were categorised as normal or at risk. Of these 111 participants, prognosis was normal or favourable for 56 (51%) using ultrasonography and for 76 (69%) using iuMRI (difference in specificity 18%, 95% CI 7 to 29; p=0·0008). No statistically significant difference was seen in infants with abnormal outcome (difference in sensitivity 4%, 95% CI −10 to 19; p=0·73).

**Interpretation:**

iuMRI remains the optimal tool to identify fetal brain abnormalities. It is less accurate when used to predict developmental outcome, although better than ultrasonography for identifying children with normal outcome. Further work is needed to determine how the prognostic abilities of iuMRI can be improved.

**Funding:**

National Institute for Health Research Health Technology Assessment programme.

## Introduction

The MERIDIAN study^1^ showed that in utero MRI (iuMRI) detects fetal brain abnormalities more accurately than ultrasonography alone.^2^ iuMRI also improves diagnostic confidence and provides additional useful clinical information in 49% of women, which changes prognosis in 24%.^1^ Subsequent work in smaller cohorts of women^3^, ^4^ and a meta-analysis^5^ have also confirmed the superiority of iuMRI over ultrasonography, although the numbers of additional abnormalities detected by iuMRI were smaller than the MERIDIAN study.

The differences in detection are likely to reflect local antenatal care practices, including whether ultrasonography was done abdominally or transvaginally, and the MERIDIAN study reflects the role of iuMRI in UK clinical practice, where transvaginal ultrasonography is not routinely done in all pregnant women.

The outcome reference diagnoses (ORD) used in the MERIDIAN study were either post-mortem results or neuroimaging done in the first 6 months of life, which was predominately cranial ultrasound. It is possible the neuroimaging ORD were incorrect or incomplete, and there is little published data to show how accurately ultrasonography and iuMRI provide prognostic information on developmental outcomes.

Understanding the accuracy of these methods is important because women and their families want to know the significance of any abnormalities found antenatally.^6^, ^7^, ^8^ The MERIDIAN 3-year follow-up study was designed to address these knowledge gaps across three projects, specifically: project one was designed to recalculate the diagnostic accuracy of ultrasonography and iuMRI using diagnoses collected after 6 months of age; project two, to compare the ability of ultrasonography and iuMRI to predict developmental outcome around age 3 years; and project three, to study the developmental significance of isolated mild ventriculomegaly (IMVM), because of the large numbers of cases and perceived difficulties in antenatal prognostication in this cohort.

**Research in context**

**Evidence before this study**

In utero MRI (iuMRI) is increasingly used in antenatal care when ultrasonography detects a fetal brain abnormality. The MERIDIAN study showed that iuMRI has better diagnostic accuracy than ultrasonography, obtaining the correct diagnosis in 93% of cases compared with postnatal imaging before age 6 months or post-mortem findings. Obstetricians reported that the results of iuMRI provided information that improved their diagnostic confidence and directly informed choices about the care of half of the pregnant women whose fetus had a suspected brain abnormality on ultrasonography. There is limited evidence on how iuMRI relates to postnatal imaging after age 6 months, and its ability to predict developmental outcome in infancy. We searched PubMed from inception to Oct 11, 2019, with no language restrictions. Search terms for in utero imaging or fetal brain abnormalities included “Fetus [MESH] AND magnetic resonance imaging [MESH]”, “Pregnancy [MESH] AND magnetic resonance imaging [MESH]”, “Prenatal ultrasonography [MESH]”, “In-utero MRI [title/abstract]”, “In-utero Magnetic resonance imaging [title/abstract]”, “Fet* magnetic resonance imaging [title/abstract]”. “Fet* imaging [title/abstract]”, “Prenatal magnetic resonance imaging [title/abstract]”, “Prenatal MRI [title/abstract]”, “Prenatal imaging [title/abstract]”, “Fet* brain abnormalit*[title/abstract]”, “Fet* brain disorder [title/abstract]”. Search terms for child development included “Child development [MESH]”, “Developmental disabilities [MESH]”, “Development* outcome [title/abstract]”, “Developmental impairment [title/abstract]”, “Developmental delay [title/abstract]”, “Child development [title/abstract]”. These two strategies were then combined using the AND function. Numerous articles showed the superiority of iuMRI to detect brain abnormalities compared with prenatal ultrasonography, including the MERIDIAN study. No article has directly compared the prognostic abilities of prenatal ultrasonography and iuMRI with reference to child development. The articles studying developmental outcome look at a small number of fetal conditions, such as ventriculomegaly or agenesis of the corpus callosum, but all articles of developmental outcome either included small numbers of participants or reviewed outcome early in childhood or without formal developmental testing.

**Added value of this study**

This study provides follow-up data on children diagnosed with a brain abnormality in utero, highlighting its diagnostic accuracy: it is rare for iuMRI to miss brain abnormalities that are visible on postnatal imaging after age 6 months. Its ability to prognosticate developmental outcome is less clear. Our results suggest that iuMRI is better at identifying children with normal outcome compared with ultrasonography, possibly by moving fetuses with an intermediate prognosis on ultrasonography to a more accurate favourable or normal prognosis. However, the developmental scores in our cohort were similar between both ultrasonography and iuMRI and also between prognosis groups, highlighting that both imaging modalities have their limitations.

**Implications of all the available evidence**

iuMRI detects brain abnormalities with high diagnostic accuracy, confirming its role in antenatal care, and there appears to be little additional value in routine postnatal imaging, given the chance of changing the diagnosis is small. Although obstetricians report that the information obtained from iuMRI often changes the information they give pregnant women, both ultrasonography and iuMRI are poor predictors of outcome. Clinicians providing antenatal counselling should be aware of this fact when offering prognostication, and avoid overconfidence when discussing the developmental significance of brain abnormalities. This is true for significant structural abnormalities and of more subtle changes, such as isolated mild ventriculomegaly, that are usually associated with good outcome. It remains to be seen to whether the accuracy of prognostication improves when paediatricians with experience following up children with neurological and developmental abnormalities are involved in multidisciplinary team discussions about the significance of ultrasonography and iuMRI results.

## Methods

### Study design and participants

The MERIDIAN 3-year follow-up study was an extension of the original MERIDIAN study, in which all families were asked to provide written consent to be contacted about future studies. Ethical approval was obtained and it was overseen by a trial steering committee. Section 251 confidentiality advisory group approval was obtained for use of the Health & Social Care Information Centre Patient Tracking system to ensure potentially eligible children were alive before approach. If the child had died, the date and cause of death were obtained but no contact was made with the family. Children were excluded if they were no longer in the care of the biological mother or if the mother was unable to understand English and no family member could translate. A letter of invitation, consent form, and a developmental screening questionnaire (the Ages and Stages Questionnaire 3rd edition;^9^ ASQ3) were sent to the families of eligible children. Families could consent for either project one or all projects. The full protocol is available online.

### Procedures

Families who consented to their child's participation in project one agreed to a review of their child's case notes and neuroimaging reports. If the latest report did not match the original ORD, or if an ORD study had not been done within the first 6 months of life, a paediatric neuroradiologist (DJAC) reviewed the neuroimaging report. As in the original study,^1^ cases in which the iuMRI was done more than 2 weeks after ultrasonography were excluded. Families who consented to project one could also return the ASQ3, even if they did not consent to project two. Families who consented to project two were invited to a developmental assessment using the Bayley Scales of Infant and Toddler Development 3rd edition^10^ (BSID3), a self-report questionnaire of the Gross Motor Function Classification Score^11^ (GMFCS), and the Strengths and Difficulties questionnaire^12^ (SDQ). If families were not willing to attend, or if the child was older than age 42 months, they were asked to complete the questionnaires only. The results of project two were combined with ASQ3 results.

Data on developmental outcome from the medical records, questionnaires, and the BSID3 assessments were reviewed independently by a paediatric neurologist (ARH) and a neonatologist (NDE), both of whom were masked to the ultrasonography iuMRI results, and ORD. Participants were categorised as normal, at risk, or abnormal (table 1). The results of the BSID3 took precedent over the ASQ3. Disagreement on developmental outcome was resolved by consensus discussion. For borderline cases, the results of the SDQ were taken into account, with a score of more than 16 taken to be abnormal (SDQ scale 0–40).

**Table 1:** Definitions used in the MERIDIAN 3-year study

	**Definition**
**Statistical definitions used for diagnostic accuracy in surviving infants**	
Specificity	100 × (number of cases with normal or at risk development AND favourable or normal prognosis/number of cases with normal or at risk development).
Sensitivity	100 × (number of cases with abnormal development AND poor prognosis/number of cases with abnormal development).
Negative predictive value	100 × (number of cases with normal or at risk development AND favourable or normal prognosis/number of cases with favourable or normal prognosis).
Positive predictive value	100 × (number of cases with abnormal development AND poor prognosis/number of cases with poor prognosis).
**Definitions used when assessing the ability to predict in-utero death, stillbirth or death during infanthood**	
Sensitivity (mortality)	100 × (number of deceased participants with poor prognosis/number of deceased participants). No other attempts were made to incorporate deaths into measures of positive predictive value, negative predictive value, or termination of pregnancy into measures of prognosis.
**Definitions used for developmental outcome**	
Normal	All BSID3 scores above 85 and no diagnosis of cerebral palsy OR; if BSID3 not done, where all ASQ3 scores were above the cut-off point and no diagnosis of cerebral palsy OR; if BSID3 and ASQ3 data not available but information from clinical notes indicated normal developmental outcome.
At risk	Any BSID3 scores between 70 and 85 OR; if BSID not done where two or more ASQ3 scores were near the cut off-level but above the cut-off level in all other domains OR; the child had cerebral palsy but motor abilities on the BSID3, or the ASQ3 were normal and GMFCS 1 or both.
Abnormal	One or more BSID3 composite score below 70 OR; if BSID not done, where ASQ3 scores were below the cut-off level in two or more developmental domains OR; ASQ3 scores were below the cut-off level in one domain and near the cut-off level in two or more domains; OR; the child had cerebral palsy with GMFCS of 2 or greater.

BSID3=Bayley Scales of Infant and Toddler Development 3rd Edition. ASQ3=Ages and Stages Questionnaire 3rd Edition. GMFCS=Gross Motor Function Classification System.

In the MERIDIAN study,^1^ obstetricians independently provided a prediction of the fetus' developmental outcome on the basis of the ultrasonography and iuMRI results as normal (chance of abnormal development no greater than the general population), favourable (≤10% chance of abnormal development), intermediate (10–50% chance of abnormal development), poor (>50% chance of abnormal development), or unknown.

In project three, the developmental outcomes of children with antenatal diagnosis of IMVM were reviewed. We included all participants in this analysis, irrespective of the time between imaging, because we were not comparing ultrasonography to iuMRI.

### Statistical analysis

The original MERIDIAN sample size calculation was applicable for project one because the 6-month diagnosis was retained unless additional diagnostic information was obtained. For projects two and three, the sample size was constrained by the number of MERIDIAN participants whose families consented to further study. The sample size for project two was informed by the number of fetuses whose prognosis changed as a result of the iuMRI (n=312), of whom a further 63 were reclassified as the poorest prognosis, and assumed approximately 400 surviving infants. Scaling these prevalences down, 400 infants would provide 90% power to detect a 20% increase in sensitivity and a 10% increase in specificity at a two-sided significance level of 5%. For project three, with approximately 140 cases and assuming poor outcome was less than 10%, allowing estimation of the prevalence to within a standard error of 2·5%.^13^

For project one, the updated imaging was used to recalculate diagnostic accuracy. Developmental outcome in relation to ultrasonography and iuMRI was summarised by sensitivity, specificity, positive predictive value (PPV), and negative predictive value (NPV; table 1). Specificities and sensitivities of ultrasonography and iuMRI were compared using the McNemar paired sample χ^2^ test, with the difference and 95% CIs calculated using the Wilson score method. We studied the ability of ASQ3 to diagnose abnormal development on BSID3, to assess whether it was appropriate to include participants with only ASQ3 results in our analysis. Sensitivities and specificities were calculated. All statistical analyses were done using R, version 3.5.2, and Stata 15.

### Role of the funding source

The funders had no role in study design, data collection, data analysis, data interpretation, or writing of the Article. The final author had full access to all the data in the study and had final responsibility for the decision to submit for publication.

## Results

**Figure 1:**
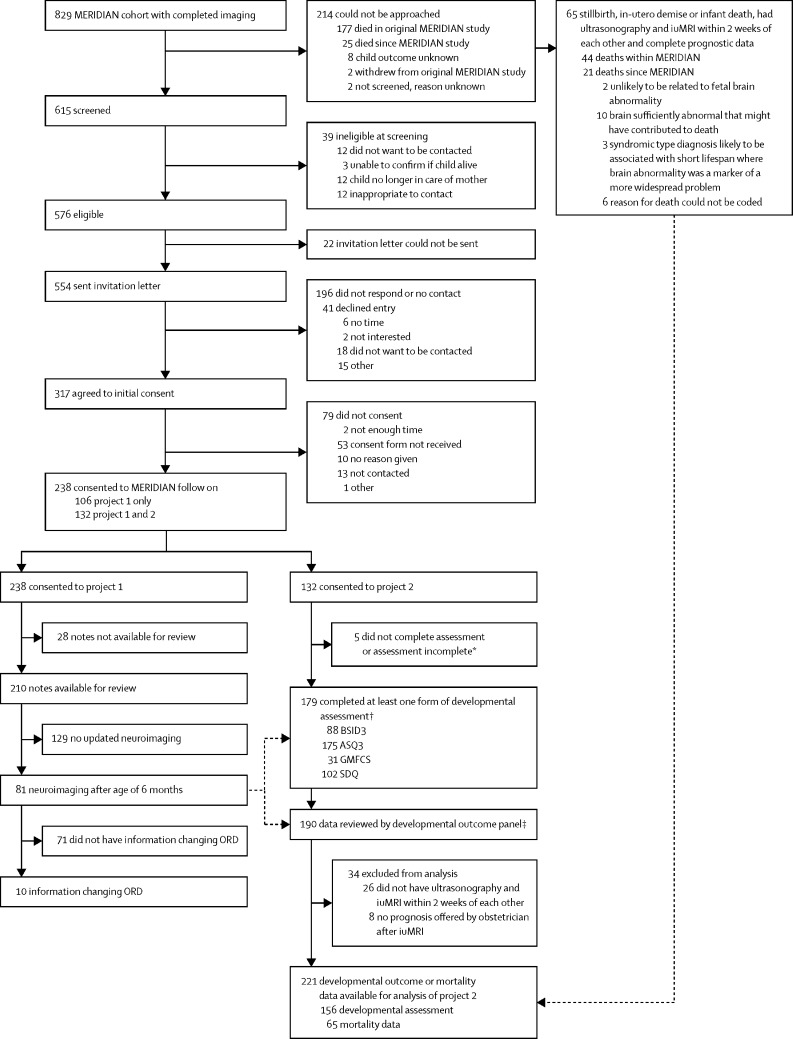
Study profile ASQ=Ages and Stages Questionnaire. ORD=outcome reference diagnoses. BSID3=Bayley Scales of Infant and Toddler Development 3rd Edition. ASQ3=ASQ 3rd Edition. GMFCS=Gross Motor Function Classification Score. SDQ=Strengths and Difficulties questionnaire.*Did not complete assessment: participants who consented, but then either did not attend or cancelled; assessment incomplete: participants who attended, but the whole assessment could not be completed or a developmental score obtained. †52 completed ASQ as part of project 1 and were included in project 2. ‡11 had developmental outcome available in medical notes and were included in project 2.

The flow of the 829 participants from the original MERIDIAN study into each of the three projects is shown in figure 1. The characteristics of the women involved in the study are summarised in the appendix (p 1). The families of 238 children consented to project one. Case notes were available for review in 210 of 238 children, of whom 81 (39%) had further investigations between age zero and 42 months (median 4 months), most commonly by postnatal MRI (n=38) or clinical assessment (n=34). Data on the reasons for the further investigations were not collected.

The ORD was unchanged in 71 (88%) of 81 cases. In five (6%) of 81 cases, the more recent ORD disagreed with the original ORD, and in a further five cases (6%) the post-6 month neuroimaging studies provided the only ORD (table 2). One participant had iuMRI more than 2 weeks after ultrasonography and was excluded from the recalculation. The recalculated diagnostic accuracies for ultrasonography and iuMRI, based on the refreshed ORD from 574 cases in total, were similar to those in the MERIDIAN study and the statistically significant difference in diagnostic accuracy was maintained (absolute difference 25%, 95% CI 21–29; p<0·0001; table 3).^13^ Developmental outcome data were available for 190 participants, of whom 164 had iuMRI within 2 weeks of ultrasonography (appendix p 3). Eight participants did not have a prognosis recorded after ultrasonography or iuMRI because the clinician did not record this at the time of counselling, leaving 156 participants who had been given an antenatal prognosis by obstetricians for analysis.

**Table 2:** The ten cases with updated outcome reference diagnosis

	**Ultrasonography diagnosis**	**iuMRI diagnosis**	**Outcome reference diagnosis at age 6 months**	Outcome reference diagnosis at age 3 years*
1	Ventriculomegaly, hypoplastic cerebellar hemispheres	Ventriculomegaly	Ventriculomegaly	Rhombencephalosyapsis
2	Ventriculomegaly	Ventriculomegaly	Ventriculomegaly	Ventriculomegaly, subependymal heterotopia
3	Ventriculomegaly, hypogenesis of corpus callosum	Ventriculomegaly	Ventriculomegaly (due to hydrocephalus)	Tectal plate tumour, hydrocephalus due to aqueduct stenosis, subependymal heterotopia
4	Hypoplastic cerebellar hemispheres	Hypoplastic cerebellar hemispheres	Hypoplastic cerebellar hemispheres	Hypoplastic cerebellar hemispheres, subependymal heterotopia
5	Agenesis of corpus callosum	Hypogenesis of corpus callosum	Hypogenesis of corpus callosum	Hypogenesis of corpus callosum, cyst of the quadrigeminal cistern, polymicrogyria
6†	Ventriculomegaly, hypogenesis of corpus callosum	Ventriculomegaly, hypogenesis of corpus callosum	Not done	Ventriculomegaly
7	Agenesis of corpus callosum	Agenesis of corpus callosum Polymicrogyria	Not done	Agenesis of corpus callosum, polymicrogyria
8	Absent cerebellum	Ventriculomegaly, Chiari II malformation	Not done	Ventriculomegaly, Chiari II malformation
9	Ventriculomegaly, agenesis of corpus callosum	Ventriculomegaly	Not done	Ventriculomegaly
10	Hypoplastic cerebellar vermis	Posterior fossa cyst	Not done	Dandy-Walker spectrum abnormality

iuMRI=in-utero MRI.

* In all cases the outcome reference diagnosis at 3 years was determined using MRI.

† This case was not included in final analysis because iuMRI occurred more than 2 weeks after the ultrasonography.

**Table 3:** Updated diagnostic accuracy of ultrasonography and iuMRI by gestational age of fetus

	**Meridian**		**Meridian 3-year follow-up**			
	Ultrasonography correct	iuMRI correct	Ultrasonography correct	iuMRI correct	Percentage difference (95% CI)	p value*
18–23 weeks (n=372)	256/369 (70%)	341/369 (92%)	257/372 (69%)	339/372 (91%)	22% (17–27)	<0·0001
≥24 weeks (n=202)	129/201 (64%)	188/201 (94%)	130/202 (64%)	190/202 (94%)	30% (23–37)	<0·0001
Combined (n=574)	387/570 (68%)	529/570 (93%)	387/574 (67%)	529/574 (92%)	25% (21–29)	<0·0001

Data are n/N (%), unless otherwise indicated. The 574 fetuses include the 570 fetuses included in the original MERIDIAN primary analysis (had iuMRI within 2 weeks of ultrasonography and had outcome reference diagnosis). There were four further cases that now have an outcome reference diagnosis from the 3-year data, increasing the number to 574. Four of the original cases also had updated outcome reference diagnosis from the 3-year data. The updated diagnostic accuracy is based on new neuroimaging studies up to age 3 years and presented as a percentage along with the percentages correct from the original MERIDIAN study for reference. iuMRI=in-utero MRI.

* McNemar's test between ultrasound and iuMRI correct diagnoses.

**Figure 2:**
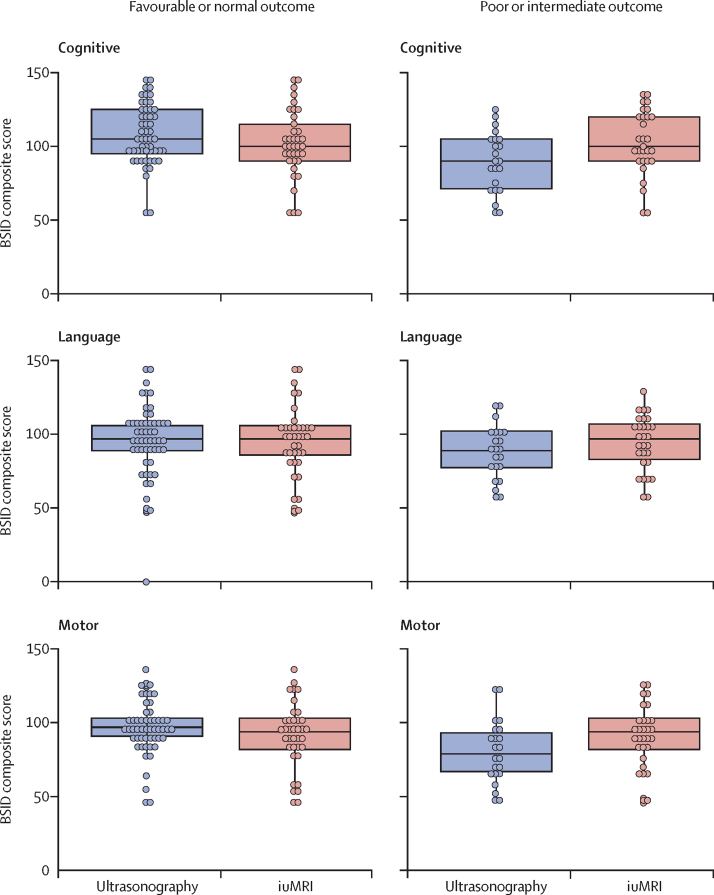
Developmental outcome for cognition, language, and motor skills of iuMRI and ultrasonography by prognosis^13^ The box represents the first (bottom) and third (top) quartiles, the line through the centre of the box represents the median. The lower whisker extends from the smallest observation greater than or equal to the first quartile minus 1·5 × IQR and the upper whisker extends to the largest observation less than or equal to the third quartile plus 1·5 × IQR. iuMRI=in-utero MRI.

The BSID3 scores were similar between imaging modality and prognosis groups in all developmental domains (figure 2). 111 (71%) of 156 infants had developmental outcomes considered normal or at risk—of these infants, 56 (50%) of 111 had a normal or favourable prognosis on ultrasonography, compared with 76 (68%) following iuMRI (difference in specificity 18%, 95% CI 7–29; p=0·0008).

No statistically significant differences were seen in the proportions of fetuses who died or who had abnormal development in childhood after being given a poor prognosis after ultrasonography or iuMRI (table 4).

**Table 4:** Outcomes of infants based on in-utero ultrasonography and iuMRI prognosis where imaging was done within 2 weeks of each other, along with specificities, sensitivities, PPV and NPV

		**Developmental assessment (surviving infants)**		**In-utero death, stillbirth or infant death (n=65)**
		Normal or at risk (n=111)	Abnormal (n=45)	
**Ultrasonography**				
Normal or favourable (≤10%)		56	13	11
	Specificity	50% (41–60)	..	..
	NPV (surviving infants only)	81% (70–89)	..	..
Intermediate (11–50%)		28	11	15
Poor (>50%)		5	9	27
	Sensitivity	..	20% (11–34)	42% (30–54)
	PPV (surviving infants only)	..	64% (39–84)	NA
Unknown		22	12	12
**iuMRI**				
Normal or favourable (≤10%)		76	19	10
	Specificity	68% (59–76)	..	..
	NPV (surviving infants only)	80% (71–87)	..	..
Intermediate (11–50%)		20	12	16
Poor (>50%)		5	11	31
	Sensitivity	..	24% (14–39)	48% (36–60)
	PPV (surviving infants only)	..	69% (44–86)	NA
Unknown		10	3	8

Data are n or % (95% CI). Prognosis following in-utero scan (chance of abnormal development as assigned by clinician). NA=not applicable. iuMR=in-utero MRI. NPV=negative predictive value. PPV=positive predictive value.

The results for all children, irrespective of the time between ultrasonography and iuMRI, were similar (appendix p 4). The proportion of children in each prognostic category who had either abnormal or combined abnormal and at risk outcomes are shown in table 5. Normal or favourable prognostic groups from ultrasonography and iuMRI data were associated with abnormal outcomes in approximately 20% of cases (ultrasonography 18 of 85 cases; iuMR 21 of 109 cases), and either abnormal or atypical outcome in around 35% of cases (ultrasonography 30 of 85 cases; iuMR 38 of 109 cases). A normal prognosis was associated with normal outcome in around 65% of cases (ultrasonography 55 of 85; iuMR 71 of 109).

**Table 5:** Prognosis estimated by fetal medicine unit based on ultrasonography and iuMRI for all participants, irrespective of the time between ultrasonography and iuMRI

	**Prognosis based on ultrasonography**		Prognosis based on iuMRI*	
	Proportion with abnormal development postnatally	Proportion with abnormal or at-risk development postnatally	Proportion with abnormal development postnatally	Proportion with abnormal or at-risk development postnatally
Normal or favourable (≤10%)	18/85 (21%)	30/85 (35%)	21/109 (19%)	38/109 (35%)
Intermediate (11–50%)	14/49 (29%)	20/49 (41%)	15/36 (42%)	20/36 (55%)
Poor (>50%)	11/16 (69%)	11/16 (69%)	13/18 (72%)	15/18 (83%)
Unknown (not known)	15/40 (38%)	23/40 (58%)	5/16 (31%)	7/16 (44%)

iuMRI=in-utero MRI.

* The decreased number of iuMRI relates to 11 who had no prognostication information offered by the obstetrician.

19 (22%) of 86 participants diagnosed with IMVM had abnormal development and 12 (14%) of 86 participants were considered at risk.^13^ None of the children received a poor prognosis category on antenatal imaging with either modality. Abnormal outcome was less likely in cases of resolved IMVM than persisting IMVM: IMVM resolved in 62 (72%) of 86 participants, nine (15%) of whom had abnormal outcome, and was persistent in 24 (28%) of 86 participants, ten (42%) of whom had abnormal outcome. 15 (17%) of 86 children had postnatally diagnosed structural or genetic abnormalities or significant life events, and these were proportionately more common in children with abnormal (seven [37%] of 19) and at-risk (four [33%] of 12) development compared with normal outcome (four [7%] of 54; table 1). 84 participants had both ASQ3 and BSID3 (appendix p 5) irrespective of the time between iuMRI and ultrasonography. The sensitivity of the ASQ3 was 90%, specificity 83%, PPV 68%, and NPV 97%.

## Discussion

Clinical outcomes beyond the immediate postnatal period have not been reported in studies comparing ultrasonography and iuMRI, which is important because features like the immature myelination state of the brain can hamper the diagnostic capacity of MRI before age 6 months. Our results show that postnatal imaging after age 6 months rarely changes ORD and the improved diagnostic accuracy of iuMRI is maintained.

The developmental significance of antenatally diagnosed brain abnormalities is important for parents, who use the information to decide whether they have the ability, and emotional and financial resources to care for their child.^6^, ^7^, ^8^, ^14^ Data on many fetal brain abnormalities that can be used during antenatal counselling are scarce,^15^, ^16^ and whether iuMRI improves prognostication is unclear.

The interpretation of our findings is not straightforward. Among the 111 infants with normal or at risk development, more participants had good prognosis with iuMRI compared with ultrasonography, suggesting iuMRI is better at excluding abnormal developmental outcome than ultrasonography, either through better visualisation of the abnormalities or identification of false positives on ultrasonography. The percentage of infants with normal or at risk developmental outcome after a good iuMRI prognosis was similar to a good prognosis on ultrasonography. The discrepancy between specificity and NPVs is likely to be secondary for two reasons. First, iuMRI was associated with fewer unknown or intermediate prognoses than ultrasonography, often moving the fetus into normal, favourable, or poor prognosis groups.

Consequently, although the NPVs were similar, iuMRI had improved prognostic accuracy compared with ultrasonography. This observation might be because iuMRI delineates or excludes other abnormalities better than ultrasonography, but there might also be a psychological factor, whereby iuMRI gives the clinician more confidence to move from an indeterminate to either a good or poor prognosis.

When the final prognosis based on iuMRI was normal or favourable, this was often correct. The second reason relates to the effect of non-uptake of the 3-year developmental assessment. Although the reasons for non-participation are speculative, fetal and infant mortality (excluding termination of pregnancy) were marginally lower after a favourable or normal prognosis from iuMRI than ultrasonography, and higher following a poor iuMRI prognosis than a poor ultrasonography prognosis. Similar patterns were seen for frequency of pregnancy terminations. Therefore, although iuMRI was superior to ultrasonography for identifying children with normal development, the two modalities were similar in their ability to predict abnormal development.

A further consideration relates to how we defined developmental outcomes. The mean population score of the BSID3 is 100 (SD 15), so around 95% of the normal population lie between 70 to 130.^10^ These scores are in keeping with the definition of early developmental impairment—in which developmental abilities have to fall two standard deviations below the population mean in two or more domains^17^, ^18^—and the International Statistical Classification of Diseases, 10th revision, classification of mild learning disabilities when an intelligence quotient (IQ) is 50–69, moderate when 35–49, severe when 20–34, and profound when below 20·^19^ By contrast, preterm follow-up studies, such as the EPICure Study,^20^ define BSID3 scores between 70 and 85 as mild impairment, 55 and 70 as moderate, and below 55 as severe, or cognitive or language scores below 85 as moderate to severe impairment.^21^

Using these definitions for fetal follow-up studies comes with challenges; for example, a single point could be the difference between a normal and moderate to severe impairment and might result from the child's tiredness, boredom, or refusal. The definitions take little account of the child's genetic or social potential: a child with a cognitive composite score of 84 whose parents' IQs are also in the 80s might be appropriate, whereas a child with a score in the normal range at 88 whose parents' IQs are 130–140 might not.

Developmental assessments at 2–3 years are imperfect predictors of later outcome,^22^, ^23^, ^24^, ^25^ because children scoring normally in the early years might exhibit significant difficulties later, and children with low scores might catch up to their peers. Therefore, care is needed, especially because terminology like severe or abnormal strongly influence patient choice,^26^, ^27^, ^28^ and we do not wish to inflate the risk of developmental problems and inadvertently coerce women towards terminating fetuses who would be normal.

Another issue was the proportion of participants assessed only with the ASQ3, which can yield false-positive results.^29^, ^30^, ^31^ A number of children who were identified by the ASQ3 as borderline or abnormal, scored normally on the BSID3 (appendix p 5). For these reasons, we chose the term at risk rather than mildly abnormal or borderline because these children might have normal outcome in the long term. We were similarly careful not to classify all participants with cerebral palsy as abnormal. This might seem a controversial choice, but women report that health-care professionals focus too much on medical diagnoses and terminology during antenatal counselling, when what they really want to know is the best and worst case scenario and the likely functional outcome of their baby.^32^, ^33^, ^34^

The views are consistent with a WHO report on disability,^35^ which notes that generalisations about disability are misleading because many people with a disability do not consider themselves unhealthy or to have a bad quality of life. WHO suggests clinicians' focus should be directed less towards medical diagnosis and more towards how it impairs an individual, affecting activity, participation, and independence. With this in mind, categorising a child with mild cerebral palsy—whose GMFCS scores are low and whose motor scores on BSID3 and ASQ3 are normal—as severely abnormal is inappropriate, particularly given they are likely to have normal quality of life.^36^ Therefore, we categorised functionally able children with cerebral palsy as at risk.

Although the number of participants in project two was lower than anticipated, our findings indicate that, despite the increase in diagnostic accuracy and confidence provided by iuMRI, it remains difficult to predict clinical outcomes on an individual basis. This result is unsurprising because prognostication data from antenatal studies are based on population studies rather than individualised risks for specific fetuses, and the effect of a given brain abnormality can vary markedly between individual infants.

A more appropriate way to analyse project two might be on a grouped basis: the data shown in table 5 indicate that if a poor prognosis is made on the basis of ultrasonography data alone, 69% of those fetuses have an abnormal outcome; the results using iuMRI information are similar (72%). These figures are consistent with the more than 50% chance used in our definition of poor prognosis. An intermediate prognosis based on ultrasonography data is associated with the chance that 29% have an abnormal outcome, whereas an intermediate prognosis on iuMRI is associated with a higher chance of an abnormal outcome (42%). Both fall into the 11–50% range used to define intermediate prognosis.

Fetuses given an unknown prognostic category had clinical outcomes similar to the intermediate category, with abnormal outcomes in 38% from ultrasonography information and 31% based on iuMRI information. The results from the normal and favourable categories showed the widest discrepancy when compared with the predicted abnormal risk rates.

The predicted risk of an abnormal outcome when those two groups are merged is 10% or less but the observed abnormal outcome rates were 21% for ultrasonography and 19% for iuMRI-derived data. This finding was unexpected and concerning because they are double the expected percentage of abnormal outcomes in cases which, presumably, had normal or minor findings on ultrasonography and iuMRI. Further analysis is planned to study the nature of the abnormalities associated with abnormal outcomes.

Other possible explanations exist for the modest correlation between the prognosis and developmental outcome. One is the lack of high-quality developmental outcome data after fetal diagnosis of neurological abnormalities. A second is that there might be other factors influencing an obstetrician's prognostication, such as maternal health, family history, fetal growth, or amniotic fluid volume, or where a combination of fetal abnormalities are found. As such, counselling must be tailored to an individual's situation, and the MERIDIAN study cannot analyse the subtleties of a clinician's thoughts or hunch. A third is the experience of the fetal medicine expert in interpreting the significance of paediatric neuroimaging: a survey of fetal medicine consultants done during the MERIDIAN study showed a wide range of prognostic grades would be given for the same antenatal imaging findings.^37^

Further research is needed to determine whether a paediatrician with experience in following up children with neurodevelopmental abnormalities might interpret antenatal imaging findings differently and provide better prognostication.

In many ways, the limitations of ultrasonography and iuMRI to prognosticate should not be surprising: most children with early developmental impairment have normal neuroimaging,^38^, ^39^ and it is common for children with abnormalities on neuroimaging to do better developmentally than expected.

In the future, gene exome work might help with prognosis, but it is unlikely to give clinicians all the information they want. For example, the PAGE study^40^ of 610 fetuses with structural abnormalities found a diagnostic genetic result in only 8·5% and a result of uncertain clinical significance in 3·9%. The challenges of delivering exome studies in a timely manner in antenatal care also need to be overcome, along with explaining to families the problem of phenotypical variation between individuals with the same mutation, even within a single family.^41^, ^42^

Project three looked at children who had IMVM. Most children with this antenatal diagnosis do not show adverse developmental outcomes, so fetal maternal clinicians usually give a favourable prognostic category. Specifically, just over half of the fetal maternal clinicians referring into the MERIDIAN study give a 90% chance of a normal outcome and the others 95% normal outcome (ie, all have >90% change of normal outcome).^37^

Large studies and meta-analyses suggest abnormal developmental outcome rates in IMVM are 5·6–12%,^43^, ^44^, ^45^ although studies differ in how IMVM is defined, with some studies using 10–11 mm and others 10–15 mm. Furthermore, some studies only consider a fetus as having IMVM when the karyotype and congenital infection screen is negative, others rely purely on the absence of other structural abnormalities on imaging.

We took a pragmatic approach because women might refuse karyotyping if they considered the risk of miscarriage related to amniocentesis or chorionic villus sampling too high. We also reviewed the medical notes and diagnoses of children for this study, and would have recognised children with genetic conditions diagnosable by karyotype antenatally and would have excluded these, but no such cases were identified.

The results of our study show the risk of an abnormal outcome was much higher at 22%, and a further 14% were considered at risk. The high prevalence of mild ventriculomegaly cases in MERIDIAN might partially explain the high proportion of abnormal outcomes in fetuses given normal or favourable prognostic categories, with selection bias also potentially playing a role because parents with concerns about their child's development might have been more likely to enrol.

The false-positive rate of the ASQ3 might also play a role. We found a higher rate of abnormal outcome when mild ventriculomegaly was associated with other abnormalities, such as somatic malformations or chromosomal abnormalities, and the risk of adverse developmental outcomes was lower when ventriculomegaly resolved in utero, which confirms previous data.^44^

The key limitation in the MERIDIAN 3-year follow-up was the low level of participation, with just under a third of eligible parents giving consent to developmental assessment. The effect of this is difficult to predict, but is likely to be non-random: previous work suggests that some parents choose not to participate in research because the burden outweighs the benefit,^46^, ^47^ which in this study might arise either for children with no obvious problems or for those with clearly abnormal development who usually require multiple other hospital appointments. This reduces the statistical power and precision, but more importantly might have led to either an over-estimation or an under-estimation of the incidence of abnormal development.

We cannot be more specific without knowing the reasons parents chose not to participate. By contrast, any selection effect is less likely to affect the direct comparison between iuMRI and ultrasonography, particularly the sensitivity and specificity for predicting abnormal development.^48^ We also were not able to administer BSID3 to all participants, relying on the ASQ3 in a proportion. Direct comparison between the two tests shows good agreement, but false positives on the ASQ3 might have inflated the abnormal and at-risk groups.

Finally, we limited the analysis of development to surviving infants. An alternative analysis would be to incorporate both surviving and non-surviving cases using survey-based weighting to account for their differential probability for inclusion. The difficulties in interpreting these measures, in particular for cases managed by termination of pregnancy, led us to focus on outcomes conditional on the infant surviving to age 3 years. These exclusions will have underestimated the true diagnostic and prognostic ability of iuMRI, given its ability to identify severe brain conditions.

Despite a low level of participation, we confirmed that the original postnatal diagnosis remained applicable for the majority of infants at their 3-year follow-up. We did not collect information on why additional investigations were (or were not) undertaken, and it is likely that a proportion of infants had more latent complications that had either not been diagnosed or not become apparent. Despite our concerns about the veracity of ORD from neuroimaging studies before 6 months of life, the updated estimates of diagnostic accuracy of ultrasonography and iuMRI for detecting fetal brain abnormalities are exceptionally close to the original calculations and confirm the diagnostic advantage of doing iuMRI.

The findings from the clinical follow-up show iuMRI improves the confidence of the fetal medicine unit team to say a fetus will have a normal outcome, but prognostication of outcome remains similar between imaging modalities. Further work is needed to determine whether this is a problem inherent with iuMRI or whether other professionals, such as paediatricians, can help to improve prognostication.

Finally, our results indicate a much higher rate of abnormal outcomes than would be expected in fetuses with minor brain abnormalities who were estimated to have normal or favourable prognosis antenatally. This is particularly the case in fetuses with mild ventriculomegaly that does not resolve in pregnancy, and larger cohort studies are needed to determine what the exact risk is and which fetuses are at the greatest risk.
