# Supplementary material for: Accuracy of in-utero MRI to detect fetal brain abnormalities and prognosticate developmental outcome: postnatal follow-up of the MERIDIAN cohort
Source: Lancet Child Adolesc Health. 2020 Feb;4(2):131–40. doi: 10.1016/S2352-4642(19)30349-9 (PMC6988445; doi:10.1016/S2352-4642(19)30349-9)
Supplement: Supplementary appendix [file mmc1.pdf]

THE LANCET
Child & Adolescent Health

Supplementary appendix

This appendix formed part of the original submission and has been peer reviewed. We post it as supplied by the authors.

Supplement to: Hart AR, Embleton ND, Bradburn M, et al. Accuracy of in-utero MRI to detect fetal brain abnormalities and prognosticate developmental outcome: postnatal follow-up of the MERIDIAN cohort. *Lancet Child Adolesc Health* 2019; published online Nov 27. https://doi.org/10.1016/S2352-4642(19)30349-9.

Table of Contents

Supplementary table 1: Participant characteristics at entry to the MERIDIAN study 1

Supplementary table 2: Combinations of developmental assessments performed in participants in project 2 2

Supplementary Table 3: Participant characteristics and study participation where iuMR was obtained within 2 weeks of USS 3

Supplementary table 4: Outcomes of infants based on iuUSS and iuMRI prognosis, specificities, sensitivities, PPV and NPV *for all cases including those who had iuMR more than 2 weeks after USS* 4

Supplementary table 5: Comparison of development categories according to BSID3 and ASQ-3 in participants who completed both assessments (n=84)... 5

Supplementary table 1: Participant characteristics at entry to the MERIDIAN study

Demographic		Overall MERIDIAN cohort	Consented to Project 1 and Project 2	Consented to Project 1 only
**Number of participants**		829	132	106
**iuMR Site (%)**	Sheffield	531 (64)	84 (64)	78 (74)
	Birmingham	124 (15)	23 (17)	21 (20)
	Newcastle	81 (10)	9 (7)	5 (5)
	Leeds	57 (7)	5 (4)	1 (1)
	Nottingham	16 (2)	1 (1)	1 (1)
	Belfast	20 (2)	10 (8)	0 (0·0)
**Maternal Age at approach (Median [IQR])**		29·00 [24·00, 33·00]	31·00 [26·75, 35·00]	30·00 [26·00, 34·00]
**Single Pregnancy (%)**	Multiple	44 (5)	5 (4)	12 (11)
	Single	784 (95)	127 (96)	94 (89)
**Gestational Age at iuMR (Median [IQR])**		22·71 [21·43, 26·43]	23·21 [21·68, 28·75]	22·64 [21·57, 25·86]
**Gestational Age at iuMR**	<=24 weeks	531 (64)	80 (61)	70 (66)
	24+ weeks	298 (36)	52 (39)	36 (34)
**Diagnosis on iuMR (%)**	1.Ventriculomegaly	405 (49)	76 (58)	60 (57)
	2.Corpus callosum	149 (18)	21 (16)	20 (19)
	3.Posterior fossa	134 (16)	19 (14)	14 (13)
	4.Other	138 (17)	16 (12)	12 (11)
**Outcome of pregnancy (%)**	Death	53 (7)	0 (0)	0 (0)
	Livebirth	642 (78)	132 (100)	106 (100)
	Termination	124 (15)	0 (0)	0 (0)
**MERIDIAN analysis population (%)**	ORD available	570 (69)	94 (71)	83 (78)
	ORD Unavailable	175 (21)	15 (11)	14 (13)
	Excluded	84 (10)	23 (17)	9 (9)

iuMR = in utero Magnetic Resonance imaging, IQR = Interquartile range, ORD = Outcome reference diagnosis

Supplementary table 2: Combinations of developmental assessments performed in participants in project 2

Developmental assessments performed	All participants in Project 2 (%)<br><br>n=190	Participants in Project 2 who had iuMR within in 2 weeks of USS (%)<br><br>n=164
ASQ3 only	60 (32)	154 (34)
ASQ3 and BSID3	16 (8)	14 (9)
ASQ3 and SDQ	31 (16)	25 (16)
ASQ3, BSID3 and SDQ	68 (36)	56 (34)
BSID3 alone	2 (1)	2 (1)
BSID3 and SDQ	2 (1)	1 (0·6)
Medical notes only	11 (6)	10 (6)

iuMR = in utero Magnetic Resonance imaging, USS = Ultrasonography, BSID3 = Bayley Scales of Infant and Toddler Development 3rd Edition, ASQ3 = Ages and Stages Questionnaire 3rd Edition, SDQ = Strengths and difficulties questionnaire

Supplementary Table 3: Participant characteristics and study participation where iuMR was obtained within 2 weeks of USS

Prognosis based on neuroimaging	Eligible (n=511)				Ineligible (n=234)			Total
	Diagnosis and development (N=164)**	Diagnosis only (N=43)	No consent/ withdrew (N=286)	Not approached (N=18)	TOP (N=119)	Fetal or infant death (N=68)	Not approached (N=47)	(N=745)
**Prognosis on USS**								
Normal	8 (40%)	0	5 (25%)	0	3 (15%)	2 (10%)	2 (10%)	20 (100%)
Favourable (>90%)	65 (25%)	26 (10%)	123 (47%)	8 (3%)	11 (4%)	9 (3%)	16 (6%)	258 (100%)
Intermediate (50-90%)	41 (19%)	9 (4%)	88 (41%)	4 (1%)	41 (19%)	17 (8%)	12 (5%)	212 (100%)
Poor (<50%)	15 (12%)	3 (2%)	24 (20%)	4 (3%)	41 (34%)	27 (22%)	5 (4%)	119 (100%)
Not Known	35 (25%)	5 (3%)	46 (33%)	2 (1%)	23 (16%)	13 (9%)	12 (8%)	136 (100%)
**Prognosis on MR**								
Normal	11 (26%)	3 (7%)	21 (51%)	2 (4%)	1 (2%)	1 (2%)	2 (4%)	41 (100%)
Favourable (>90%)	84 (28%)	27 (9%)	148 (50%)	9 (3%)	3 (1%)	9 (3%)	16 (5%)	296 (100%)
Intermediate (50-90%)	32 (22%)	7 (4%)	60 (41%)	5 (3%)	15 (10%)	16 (11%)	10 (6%)	145 (100%)
Poor (<50%)	16 (9%)	4 (2%)	35 (19%)	1 (0%)	83 (46%)	31 (17%)	7 (3%)	177 (100%)
Not Known	13 (23%)	0	16 (28%)	0	11 (19%)	8 (14%)	8 (14%)	56 (100%)
No prognosis given	8 (26%)	2 (6%)	6 (20%)	1 (3%)	6 (20%)	3 (10%)	4 (13%)	30 (100%)

iuMR = in utero Magnetic Resonance imaging, USS = Ultrasonography, TOP = Termination of pregnancy

*Figures relate to the primary population of 745 participants whose in-utero USS and MR scans were performed within 14 days
** One participant completed the ASQ-3 but did not consent to Project 1 but has been included in this column for simplicity

Supplementary table 4: Outcomes of infants based on iuUSS and iuMRI prognosis, specificities, sensitivities, PPV and NPV ***for all cases including those who had iuMR more than 2 weeks after USS***

Prognosis *	Developmental assessment (surviving infants) Normal/at risk (N=132)	Developmental assessment (surviving infants) Abnormal (N=58)	In-utero demise, stillbirth or infant death (N=75)
**USS**			
Normal or Favourable (≤10%)	67	18	12
Specificity (95% CI)	*51% (42% to 59%)*		
NPV (surviving infants only)	*79% (69% to 86%)*		
Intermediate (11-50%)	35	14	17
Poor (>50%)	5	11	29
Sensitivity (95% CI)		*19% (11% to 31%)*	*39% (28% to 50%)*
PPV (surviving infants only)		*69% (44% to 86%)*	
Unknown	25	15	17
**iuMR**			
Normal or Favourable (≤10%)	88	21	11
Specificity (95% CI)	*67% (58% to 74%)*		
NPV (surviving infants only)	*81% (72% to 87%)*		
Intermediate (11-50%)	21	15	18
Poor (>50%)	5	13	36
Sensitivity (95% CI)		*22% (14% to 35%)*	*48% (37% to 59%)*
PPV (surviving infants only)		*72% (49% to 88%)*	
Unknown	11	5	10

iuMR = in utero Magnetic Resonance imaging, iuUSS = in utero Ultrasonography, PPV = Positive predictive values, NPV = Negative predictive values, CI = Confidence interval

* Prognosis following in-utero scan (chance of abnormal development as assigned by clinician)

Supplementary table 5: Comparison of development categories according to BSID3 and ASQ-3 in participants who completed both assessments (n=84)

		BSID3			
		Abnormal	Borderline	Normal	Total
ASQ-3	Abnormal	17	7	1	25
	Borderline	2	2	8	12
	Normal	0	3	44	47
	Total	19	12	53	84

BSID3 = Bayley Scales of Infant and Toddler Development 3rd Edition, ASQ3 = Ages and Stages Questionnaire 3rd Edition

Sensitivity of 90% (95% CI: 67, 99%), specificity of 83% (95% CI: 70, 92%), PPV 68%, NPV 97%
